# The alternative approach of low temperature-long time cooking on bovine *semitendinosus* meat quality

**DOI:** 10.5713/ajas.18.0347

**Published:** 2018-09-13

**Authors:** Ishamri Ismail, Young-Hwa Hwang, Allah Bakhsh, Seon-Tea Joo

**Affiliations:** 1 Division of Applied Life Science (BK21^+^), Gyeongsang National University, Jinju 52852, Korea; 2 Faculty of Bioresources and Food Industry, Universiti Sultan Zainal Abidin, Besut Campus, Terengganu 22200, Malaysia; 3 Institute of Agriculture & Life Science, Gyeongsang National University, Jinju 52852, Korea

**Keywords:** Sous Vide, Hanwoo Beef, *Semitendinosus*, Low Temperature-long Time, Shear Force

## Abstract

**Objective:**

This study aimed to elucidate whether innovative sous vide treatment has a significant influence on the beef *semitendinosus* muscle as compared to common sous vide treatment and traditional cooking.

**Methods:**

The innovative sous vide treatments were cooked at 45°C and 65°C for 6 h (SV45–65), common sous vide treatment at 45°C and 65°C for 3 h (SV45 and SV65) and traditional cooking at 75°C for 30 min (CON75). Water loss and cooking loss, as well as the physical properties (color and shear force) and chemical properties (protein and collagen solubility) of the treated meat, were investigated.

**Results:**

The results obtained indicated that the innovative sous vide with double thermal treatment (SV45–65) and cooked with air presence (CON75) resulted to lower a* and higher b* values, respectively. The water loss and cooking loss increased when temperature increased from 45°C to 65°C, and lower water loss was recorded in SV45 and CON75. These samples presented higher water content and revealed strong correlation to protein solubility. Warner-Bratzler shear force (SF) analysis showed the marked interaction between cooking temperature and time. Sample cooked at a high temperature (CON75) and a long period (SV45–65) showed a significantly lower value of SF than sample SV65 (p<0.05). Interestingly, there was no difference in SF values between SV45–65 and CON75.

**Conclusion:**

The innovative sous vide treatment with double thermal effect appears an attractive cooking method as compared to common sous vide and traditional cooking method, as it has a potential for improving tenderness values of cooked beef *semitendinosus* muscle.

## INTRODUCTION

The beef, roughly contains 26% to 31% of proteins [1], represent the main constituents that make up the structure of the meat product. They experience substantial structural changes during heating and thereby the quality of the meat will be altered after cooking [2]. At low temperature 40°C to 52.5°C, denaturation of sarcoplasmic and myofibrillar proteins occurs leading to a slow loss of fluid from the myofibers without shortening. Increasing temperature from 52.5°C to 60°C, cause an increasingly rapid loss of fluid from the myofibers, but still without overall shortening of the meat. However, cooking at 64°C to 94°C, increased the cooking loss due to the overall shortening by the heat shrinkage of the endomysial, perimysial, and epimysial collagen. With a long period of cooking, partial or complete gelatinization of collagen occurs resulting in the soft and tender feature of meat [3]. In this study, to allow greater control over temperature gradients as well as to minimize the damage to heat sensitive proteins, low temperature-long time of sous vide treatment was used. Sous vide cooking is defined as raw materials which are cooked under controlled temperature and time, inside heat-stable food grade vacuumized pouches [4]. This technique allows heat to be efficiently and uniformly transferred from the water to the meat.

Heat treatment of meat at low temperature over prolonged times has been studied by many researchers; on beef [5–9], pork [10–12], and lamb [13]. Commonly in the temperature and time ranges of 65°C to 90°C and 2 to 48 h, respectively. It was believed that this technique produced more benefits (increased tenderness, improved color retention, texture, and flavor) than traditionally cooked foodstuffs because of the mild process and the absence of oxygen in the pack [14]. Previous studies showed that low-temperature long-time treatment causes an increase in tenderness for beef [15–19], bulls [20], pork [21], and lamb [13]. A major benefit of using low temperature-long time cooking of meat is that it gives more uniform tenderness, particularly for non-primal cuts of meat [18].

The intramuscular connective tissue and the myofibrillar component contributes to the toughness of meat. In many cuts, connective tissue is the main reason meat is tough, but the myofibrillar proteins are sometimes dominant and this is referred to as actomyosin toughness [14,22]. The cuts high in connective tissue such as *semimembranosus*, *deep pectoralis* and *semitendinosus* can be improved by low-temperature long-time treatment [5,23]. This treatment has been suggested to affect chemical and physical properties of connective tissues [20] by the solubilization of the intramuscular collagen during an extended cooking time [24]. In this framework, the new approach of continuous cooking at two different temperature combinations deserves considerable attention as a method to give a double thermal effect on the myofibrillar and connective tissue. To the best knowledge of the authors, no studies have been conducted to deal with the double effect of thermal heating on sous vide meat quality. The aim of this study was to compare the effect of an innovative combination of temperature at 45°C and 65°C cooking technique with traditional cooking, and common sous vide treatment on *semitendinosus* of bovine muscle by means of protein solubility, collagen solubility, and physicochemical parameter evaluation.

## MATERIALS AND METHODS

### Raw materials

The Hanwoo beef muscles (*semitendinosus*) were obtained from a homogenous production batch at a slaughterhouse after 24 h post-mortem. No information regarding the pre-slaughter conditions of the animals used for the present study was available and muscles were selected randomly on the day of collection. Water content, pH, and total collagen of the raw sample at 24 h post-mortem was 73.21%, pH 5.40, and 7.13 mg/g, respectively. Muscles were then cut into four blocks (190±10 g) and were labeled as 45°C, 65°C, 45°C to 65°C, and 75°C. Each treatment was repeated twice.

### Sampling and heat treatment

Samples were divided according to heat treatment (45°C, 65°C, 45°C to 65°C, and 75°C). Sous vide treatments were first vacuum packaged (nylon/polyethylene) and carried out at three temperature points using a sous vide cooker (travellortech precision cooker immersion, US): single thermal treatment at 45°C for 3 h (labeled as SV45), and at 65°C for 3 h (labeled as SV65) and double thermal treatment at 45°C and 65°C (3 h for each temperature, labeled as SV45–65). Traditional cooking was carried out in polyethylene packaging without vacuum and cooked at 75°C in a water bath (JSWB-22T, 30L, Gongju, Korea) until the internal temperature was reached to 72°C (about 30 min). After cooking each block of the samples was placed in ice flakes for 10 min.

Samples were weighed before and after cooking to measure the percentage of cooking loss. Then, cooking loss fluid was collected for the measurement of collagen solubility. Heat-treated samples were stored in vacuum pack overnight at 4°C prior to analysis.

### Color measurement

Color determination of samples was carried out using a Konica Minolta Colorimeter (CR-300 Chroma meter, Osaka, Japan) equipped with a standard illuminant D65 using a 2° position of the standard observer with a pulse xenon lamp and 8 mm of reading surface area. The assessments were carried out on five preselected locations at the cut surface of each sample. L* (lightness), a* (redness), b* (yellowness) values were recorded.

### Water content and water loss

Water content was determined by drying the samples (4 g) at 105°C for 16 h [25]. Water loss was calculated as the percentage difference between the water content of raw and cooked samples.

### Warner-Bratzler shear force

The analysis was carried out on the cooked sample based on AMSA guidelines [26]. Sample cores (about 1 cm in diameter) were removed parallel to the myofiber. Only muscle with no visible fat and connective tissue was used for this analysis. The cores were sheared perpendicular to the myofibers orientation using the Instron tensile testing (Instron 4443, Norwood, MA, US). Peak force was obtained using 100 N loadcell tension applied at a crosshead speed of 250 mm/min. Maximum peak force recorded during the test was reported as shear force. Ten cores samples were analyzed and following exclusion of the highest and lowest values, the average of five measurements was recorded for each sample.

### Protein solubility

The protein solubility was determined according to the method describe by Joo et al [27] with slight modification. First, sarcoplasmic proteins were extracted from 1 g muscle using 20 mL of ice-cold 0.025 M potassium phosphate buffer (pH 7.2). The samples were minced, homogenized by a high-speed homogenizer (IKA, model T25D, Staufen, Germany) at the lowest speed (11,000 rpm/min). The homogenized samples were kept under refrigerated conditions for 20 h at 4°C and then centrifuged at 3,000 g for 15 min (4°C). The supernatant was decanted and protein concentration was measured using the Biuret method with bovine serum albumin as a standard. Total protein solubility was similarly determined in a 1.1 M KI, 0.1 M potassium phosphate buffer (pH 7.2). Myofibrillar protein solubility was calculated by the difference in the solubility of total and sarcoplasmic proteins.

### Total collagen and collagen solubility

Total collagen was determined according to ISO-3496 [28] with a slight modification. Four gram of meat was hydrolyzed with 30 mL of 3.5 M H_2_SO_4_ for 16 h at 105°C. The hydrolysate was filtered and the solution was diluted to volume of 500 mL with distilled water. Diluted solution 1 mL was pipetted into a 100 mL graduated cylinder and filled to mark with distilled water. Final dilution 2 mL was pipetted into a test tube and 1 mL oxidation solution (1.41 g chloramines-T reagent and 100 mL pH 6.0 buffer solutions) was added and left at room temperature for 20 min. The buffer solution was prepared by dissolving 90 g sodium acetate trihydrate, 15 g sodium hydroxide and 30 g citric acid monohydrate into 290 mL 1-propanol, and then diluted to 1 L with distilled water. Color reagent 1 mL (dissolving 10 g 4-(dimethylamino) benzaldehyde in 65 mL 2-propanol and 35 mL perchloric acid) was added and mixed. The tube was capped and then placed in a water bath at 60°C for 15 min. After cooling, the absorbance of solutions was measured at 560 nm with a UV-Vis spectroscopy (Agilent 8453, Santa Clara, CA, US). A standard calibration curve was carried out from hydroxyproline at concentration 0, 1.2, 2.4, 3.6, 4.8 μg hydroxyproline/mL. The collagen content was calculated from hydroxyproline content using the coefficient 8.

Soluble collagen in the cooking loss was centrifuged at 3,000 g, at 4°C for 30 min. Supernatant 5 mL was hydrolyzed, diluted with distilled water (first to 100 mL, then to 50 mL) and measured for hydroxypropylation concentration as described for total collagen. Collagen solubility (%, soluble collagen in the cooking loss) is expressed in Equation (1).

(1) $$\%Collagen\;solubility=\left(\frac{Soluble\;collagen\;in\;cook\;loss,g}{Total\;collagen\;of\;raw\;meat,g}\right)\times 100$$

### Statistical analysis

Data were analyzed by one-way analysis of variance as the sole source of variation and a Duncan test for multiple mean comparisons. Pearson correlation analysis was used to establish a linear relationship between measurements. All statistical analyses were performed using measurement means and standard deviation with the SPSS version 23 (IBM Corp., SPSS, Statistic, Armonk, NY, USA). Each trial was run in triplicate, except for color measurement and Warner-Bratzler shear force which were the average of five measurements.

## RESULTS AND DISCUSSION

### Instrumental color parameters

The instrumental color parameters (lightness, L*; redness, a*; and yellowness, b*) of cooked beef *semitendinosus* are reported in Table 1. The cooked samples SV65, SV45–65, and CON75 were lighter compared to SV45, but L* did not significantly differ among SV65, SV45–65, and CON75. In the present study, our results showed an increased in L* value after cooking from 45°C (SV45) to 65°C (SV65) for the same period of cooking at 3 h, while from 65°C (SV65) to 75°C (CON75) had no difference (p>0.05), at different cooking times. Determining the effect of time on changes of L* value is impossible because of inconsistent results. For example, Roldan et al [13] studied sous vide treated lamb at 60°C, 70°C, and 80°C for 6, 12, and 24 h found fluctuation in L* values in regards to time. Whilst, Sanchez Del Pulgar et al [21] found that time effect on L* values of pork samples was almost similar and Christensen et al [11] observed an only slight increase in L* values in relation to time. Thus, the L* value changes can be said influenced by the temperature rather than time and cooking method. The temperature-dependent color change relies not upon only the total amount of myoglobin and its derivatives in muscles but on the chemical status of the denatured myosin and actin as well [2]. The meat samples become lighter after heat treatment was due to the increase reflectance and scattering of light by denatured proteins and aggregated sarcoplasmic and myofibrillar proteins [11,29]. However, other authors have reported the opposite results with L* value more intense at 60°C than 80°C [21] in pork and [13] in lamb samples. Moreover, no treatment effect was observed for L* values (p>0.05) among the cooked samples after 65°C in this study.

The intensity of the a* values of cooked meat was significantly influenced by temperature and cooking time. Because a high thermal treated sample (CON75) and longer cooking time sample (SV45–65) showed a significant decrease in a* value (p<0.05) as compared to SV45 (Table 1). Accordingly, a more intense red color (higher a* values) was observed in SV45 than in SV65, SV45–65, and CON75. According to Florek et al [30], the denaturation of myoglobin starts between 55°C and 65°C, and the process is most intense between 75°C and 80°C. Again, the denaturation rate of myoglobin is reduced with increasing temperature. But this was not supported by our data for sample CON75 (cooked at 75°C), because there was no difference to samples cooked at 65°C (SV65 and SV45–65). Furthermore, sous vide in conjunction with double thermal effect and prolonged cooking time (SV45–65) was observed to have slightly decreased a* values compared to other samples in the present study. This could potentially be due to the prolonged cooking causing myosin and actin to denature as well as increasing soluble forms such as pigment heme in cooking loss [30], which do not add to the red color, but instead overrides the red color of myoglobin [31].

The least susceptible to the heat treatment proved to be deoxymyoglobin and oxymyoglobin. An increase in the cooking temperature resulted in an increase of metmyoglobin and sulfmyoglobin [17,32]. In short, a loss of reddish and a yellowish increase was observed (Table 1). CON75 displayed higher b* values than SV45–65 and SV45 but showed no difference to SV65. It is possible that oxidation to metmyoglobin can be protected by use of sous vide vacuum packaging in this study.

### Water content, water loss, cooking loss, and protein solubility

**Figure 1:**
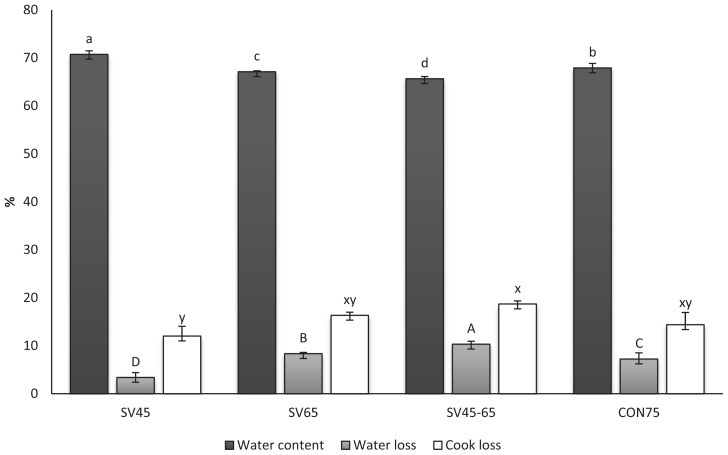
Water content, weight loss, and cook loss of *semitendinosus* from beef muscles heated at different temperature-time combinations. SV45 sous vide at 45°C (cooked for 3 h), SV65 sous vide at 65°C (cooked for 3 h), SV45–65 double thermal sous vide at 45°C and 65°C (cooked for 6 h), CON75 traditional cooking at 75°C (cooked for 30 min). Error bars represent standard deviation. The histogram bar with different letters are significantly different (p<0.05).

**Figure 2:**
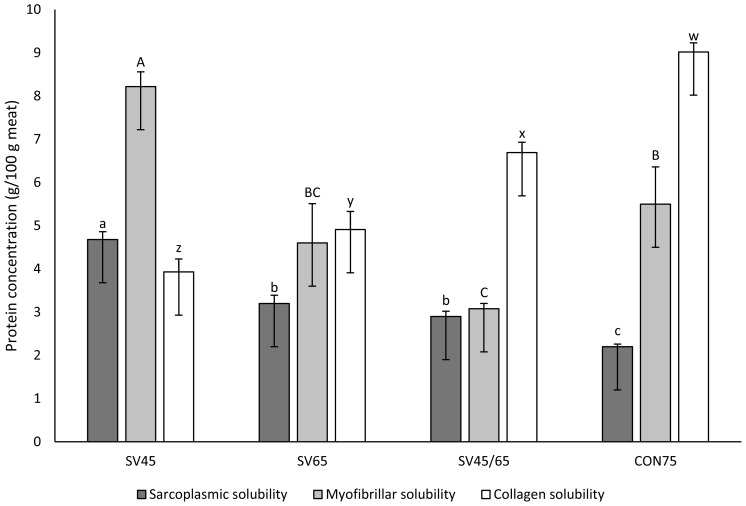
Sarcoplasmic, myofibrillar, and collagen solubility (g/100 g of meat) of *semitendinosus* from beef muscles heated at different temperature-time combinations. SV45 sous vide at 45°C (cooked for 3 h), SV65 sous vide at 65°C (cooked for 3 h), SV45–65 double thermal sous vide at 45°C and 65°C (cooked for 6 h), CON75 traditional cooking at 75°C (cooked for 30 min). Error bars represent standard deviation. The histogram bar with different letters are significantly different (p<0.05).

Water content, water loss and cooking loss for both sous vide and traditionally cooked samples are reported in Figure 1. Raw samples presented a water content of about 73% which was significantly higher than heat treated samples. There were significant differences observed between cooked samples where SV45 presented the highest value of water content (70.73%), while SV45–65 the lowest (65.66%). SV45–65 and SV65 showed significantly higher weight loss and lower water content than SV45 and CON75. Though traditionally cooked sample (CON75) was cooked at high temperature of 75°C and with the presence of oxygen, the samples in the traditional cooking method had better water retention than the sous vide samples, except for SV45. Cooking time had a significant effect on these parameters [21,33]. Thus, samples cooked for less time (traditional cooking method) showed less water loss than samples cooked for 6 h (SV45–65). Interestingly, vacuum packaged SV45 samples had slightly greater water content and lower water loss than CON75 even though they were cooked for 3 h. Therefore, it confirms that, at a lower temperature, sous vide treatment with vacuum sealed bag prevents water loss from meat samples due to the solubilized proteins (sarcoplasmic and myofibrillar proteins) binding water which results in water retention in the muscle system (Figure 2). But, increasing the temperature and time of sous vide cooking led to an increase in water loss. However, CON75 in traditional cooking could be expected to have a higher extent of water loss if the cooking time were to be increased. Because the present data evidenced that there was no significant difference in cooking loss between CON75 (cooked for 30 min) and SV45–65 (cooked for 6 h), only a 4.32% difference. While a comparison between sous vide treated samples SV45 and SV65 at an equal cooking period (3 h) clearly showed that cooking loss increased with temperature (p>0.05).

Water loss is a natural process in the meat system. It can be caused by evaporation from the surface of raw meat, or as an exudate when a muscle is cut. After a cut, the open surface of muscle fibers expresses out the liquid of sarcoplasmic proteins by gravity and this liquid is called as drip loss [2]. With regards to temperature, myofibrillar proteins start to shrink at 40°C and this process is more intense when temperature increased. In the present study, the relationship between protein solubility and retention of moisture were not overlooked. As shown in Figure 1, the high water content of sample SV45 could be due to protein solubility. According to Khan et al [34], high water holding capacity (WHC) and cooking yield are related to high protein solubility. The review by Hamm [35] indicates many of researchers have found that most sarcoplasmic proteins aggregate between 40°C and 60°C. The aggregated sarcoplasmic proteins form a gel between the structural meat elements that links them together and thereby provides consistency of cooked meat [36]. The good WHC of CON75 also can be explained by myofibrillar and sarcoplasmic proteins. The relationship between solubility of myofibrillar and sarcoplasmic proteins to water retention is frequently used to evaluate protein denaturation and its effect on water-holding capacity [37]. As shown in Figure 2, CON75 contains myofibrillar proteins with high solubility. According to Miyaguchi et al [38], meat examined under electron microscopy showed that upon heating at 70°C for 30 min the formation of network between myofibrils and sarcoplasmic proteins resulting in the formation of a condensed and fine gel as well as denatured sarcoplasmic proteins producing porous structures between denatured myofilaments, which together caused a gel matrix good at holding water. From the Pearson correlation, a strong linear correlation was obtained between water content and soluble proteins of myofibrillar and sarcoplasmic (Table 2). The results suggest that these proteins may influence both cook loss and water loss.

### Shear force and collagen solubility

The shear force (SF) of *semitendinosus* of cooked beef samples are presented in Table 1. In this study, SV45 exhibited lower SF while SV65 cooked samples presented a significantly higher toughness value. Interestingly, there was no difference in the toughness between samples SV45–65 and CON75. According to Sanchez Del Pulgar et al [21], the texture characteristics of cooked samples will depend greatly on the cooking method and collagen solubility. Collagen solubilization in mammals can be attained above 65°C [39], and Palka [40] reported that cooked beef *semitendinosus* muscles showed little soluble collagen change with core temperatures up to 60°C but it doubled at 70°C, and again drastically decreased at the range of 80°C to 121°C. Similarly, our result with CON75 cooked at 75°C showed the highest soluble collagen. Despite CON75 containing the highest soluble collagen, the SF value did not differ significantly to the SV45–65 (Table 1).

Moreover, as a comparison between sous vide sample SV65 and SV45–65 in SF value, the lower toughness of SV45–65 might be attributed to either time of cooking (6 h) or double effect of thermal (45°C and 65°C). As Roldan et al [13] stated, the cause of the changes in texture parameter as a consequence of cooking and time is very difficult to elucidate. However, many researchers have confirmed that despite the temperature, duration of cooking also has a large effect on the tenderness of meat [4,11,13,18–20,23]. Second, the combined effect of temperature at 45°C and 65°C possibly contributes to reducing toughness of the SV45–65. However, the second thermal effect at 65°C in this study seemed to be not high enough to solubilize collagen even after cooking for 6 h. In contrast, CON75 cooked for the shorter period presented the highest collagen solubility. Further investigations of cooking at two different temperatures are needed in order to elucidate possible mechanism in the tenderization process.

## CONCLUSION

Treatment temperature, duration of cooking and method of cooking (sous vide and traditional cooking) had a significant effect on quality parameters such as color, water loss, cooking loss, and Warner-Bratzler shear force as well as protein and collagen solubility. L* values were not much affected by the different treatments assayed. Prolonged heating in SV45–65 resulted to lower a* (redness) values while cooking in a bag with oxygen presence (CON75) increased b* values. A significant increase of water loss and cooking loss occurred when the temperature was increased from 45°C to 65°C. A lower of water loss in SV45 and CON75 was observed, and this linearly correlated to the protein solubility. Finally, the double thermal treatment (SV45–65) had lower SF values than single thermal effect (SV65) but no difference to CON75 (p>0.05), excluding SV45. The tenderness effect could be expected to be increased in sous vide cooked at two different temperatures if the final cooking temperature used was higher than 65°C.

## Figures and Tables

**Table 1:** Mean values of instrumental color parameters and Warner-Bratzler shear force of cooked beef1)

Items	SV45	SV65	SV45–65	CON75
Color				
L*	44.32±0.61^b^	56.06±1.53^a^	55.20±0.97^a^	56.40±1.42^a^
a*	20.12±1.13^a^	18.64±1.03^ab^	17.99±0.92^b^	18.17±1.80^b^
b*	3.81±081^c^	10.55±0.95^ab^	9.43±1.56^b^	11.19±0.23^a^
Warner-Bratzler				
Shear force (N)	31.49±0.82^c^	75.54±0.39^a^	63.57±0.61^b^	63.08±0.84^b^

1) SV45 sous vide at 45°C, SV65 sous vide at 65°C, SV45–65 double thermal sous vide at 45°C and 65°C, CON75 traditional cooking at 75°C.

Mean±standard deviation.

a–c Means with different letters within a row are significantly different (p<0.05).

**Table 2:** Pearson correlation coefficients for soluble sarcoplasmic, myofibrillar, and collagen, water content, water loss, cook loss, and color properties

Variable	Soluble sarcoplasmic	Soluble myofibrillar	Soluble collagen	Water content	Water loss	Cook loss	L*	a*	b*
Shear force	−0.71**	−0.76**	0.40	−0.78**	0.78**	0.70	0.85**	−0.56	0.82*
Soluble sarcoplasmic	-	0.57**	−0.65**	0.66**	−0.66**	−0.62	−0.96**	0.29	−0.93**
Soluble myofibrillar	-	-	−0.51*	0.85**	−0.85**	−0.84**	−0.57	0.87**	−0.48
Soluble collagen	-	-	-	−0.40	0.40	0.83*	0.77*	0.04	0.74*
Water content	-	-	-	-	−1.00**	−0.77*	−0.72*	0.77*	−0.64
Water loss	-	-	-	-	-	0.77*	0.75*	−0.76*	0.67
Cook loss	-	-	-	-	-	-	0.52	−0.66	0.48
L*	-	-	-	-	-	-	-	−0.29	0.98**
a*	-	-	-	-	-	-	-	-	−0.14

** Correlation is significant at the 0.01 level (2-tailed).

* Correlation is significant at the 0.05 level (2-tailed).
